# Graduate Students’ Perceived Supervisor Support and Innovative Behavior in Research: The Mediation Effect of Creative Self-Efficacy

**DOI:** 10.3389/fpsyg.2022.875266

**Published:** 2022-06-17

**Authors:** Jiying Han, Nannan Liu, Feifei Wang

**Affiliations:** ^1^School of Foreign Languages and Literature, Shandong University, Jinan, China; ^2^School of Translation Studies, Shandong University, Weihai, China

**Keywords:** graduate students, supervisor support, creative self-efficacy, innovative behavior, research innovation

## Abstract

With increased global competition and the advent of the knowledge economy, developing graduate students’ ability to innovate in their research has become a core focus of graduate education. Graduate students’ perceived help and assistance from supervisors is one of the key resources for research innovation. This study explored the relationships between graduate students’ perceived supervisor support and their innovative behavior in research, and examined the mediation effect of creative self-efficacy, their confidence in abilities to generate creative ideas or produce creative outcomes. Survey data were collected from a sample of 996 Chinese graduate students. The results revealed that academic support was negatively related to idea generation and idea search; personal support was positively related to overcoming obstacles; autonomy support was positively related to all factors of innovative behavior except overcoming obstacles and innovation outputs. The mediation analysis suggested that creative self-efficacy significantly mediated the relationship between academic support and graduate students’ innovative behavior in research. The results of this study highlight the significance of both supervisor support and creative self-efficacy in developing graduate students’ research innovation. The findings have significant implications for stimulating students’ research innovation and for improving the quality of graduate education.

## Introduction

Research innovation in higher education institutions (HEIs) weighs heavily in national systems of innovation (Chen and Kenney, 2007), and it plays a critical role in facilitating technological breakthroughs and economic development (Zhou, 2021). As future independent researchers and practitioners, graduate students are major sources of research innovation (Tang et al., 2020). However, enhancing the level of innovation among graduate students has become one of the most pressing problems in China’s current graduate education (Zhao, 2016), and much has been written about the intellectual and emotional challenges graduate students face in the process of innovation and the obstacles encountered by HEIs in developing innovative talents (Gu et al., 2015; Liu et al., 2019). Given the ever-increasing societal demands made on high-quality and innovative personnel, the issue of developing graduate students’ innovative behavior has gained greater attention among researchers, educators, and administrators (Acar and Tuncdogan, 2018).

Graduate students’ innovative behavior may be influenced by different contextual and individual factors (Hammond, 2016). At the contextual level, supervisor support is of key importance (Gu et al., 2015). As supervisors provide intellectual guidance, offset the risks of the research project, and help navigate the research process for graduate students, they tend to influence the process, quality, and outcomes of students’ research training, including their innovative behavior in research (Tierney and Lanford, 2016). Research has suggested that the support and guidance of supervisors significantly affect graduate students’ pursuit of innovation in their research (Fan et al., 2018). Meanwhile, creative self-efficacy, an indicator of an individual’s ability to create, is fundamental in understanding individual innovative behavior (Puente-Diaz, 2016). Large-scale workplace studies have proposed creative self-efficacy as a key motivational factor that is closely related to individual innovative behavior (Tierney and Farmer, 2011). The literature has further suggested that creative self-efficacy may not only directly contribute to innovation, but may also mediate the relationships between contextual factors and innovative behavior (Javed et al., 2020).

Although individual innovative behavior is broadly agreed to be a complex and multistage process (Montani et al., 2014), research on graduate students’ innovative behavior has mostly been based on unidimensional measures. To increase our knowledge of graduate students’ innovative behavior in research, it is necessary to examine how their perceived supervisor support is related to the multiple stages of innovative behavior in research and the mediating effect of creative self-efficacy on the relationships. Therefore, this study seeks to address the above questions based on a multidimensional construct of individual innovative behavior.

## Literature Review

### Supervisor Support

Supervisor support, the help and assistance that graduate students received from the supervisors they are assigned to Ahmed et al. (2017), is significant for graduate students’ research training (Holloway and Walker, 2000). When conducting research, supervisors offer direct and indirect support to help graduate students carry out certain tasks, such as selecting a research topic, managing the research process, and writing papers. As such, they become the main source of intellectual guidance and support for those students (González-Ocampo and Castelló, 2018). This is particularly true for Chinese graduate students since the research supervision system in China’s current graduate education is characterized by “single supervisor supervision” which requires the supervisor to take primary responsibilities for graduate students’ research training and provide adequate support and assistance for them (Peng, 2015). A growing number of studies have suggested that supervisor support is positively associated with graduate students’ research commitment, satisfaction, innovation, productivity, performance, and other outcomes (Green and Bauer, 1995; Armstrong, 2004; Dericks et al., 2019).

It is widely accepted that graduate students experience lots of hardships during the research training process (Oswalt and Riddock, 2007). Given the heterogeneity of the difficulties and obstacles that graduate students may encounter, supervisors are required to offer them different types of support and guidance (Hockey, 1996). Early research emphasized the importance of both academic and personal support and suggested that effective supervision should comprise both types (Ballard and Clanchy, 1993). Academic support refers to supervisors’ direct help with academic activities or research tasks, such as attaining the necessary research resources, planning the research design, and teaching the necessary research skills. Personal support highlights the role supervisors play in helping graduate students overcome difficulties in their research and ensuring their psychological well-being (Green and Bauer, 1995; Overall et al., 2011).

However, some researchers have argued that those two types of support are insufficient to prepare graduate students for a research career, as independent researchers possess not only a high level of academic expertise but also a great deal of confidence in their own research ability (Manathunga and Goozée, 2007). To develop highly qualified researchers and talents, supervisors must manage the balance between teaching graduate students research knowledge and developing their ability to conduct research independently (Delamont et al., 1998). Therefore, Overall et al. (2011) suggested including “autonomy support” as an extra dimension when considering supervisor support. Support for autonomy matters when supervisors encourage graduate students to make their own choices and provide them with opportunities to express ideas and conduct research independently, and it is crucial for the development of autonomous and competent researchers (Manathunga and Goozée, 2007; Gu et al., 2015).

### Individual Innovative Behavior and Its Relationship With Supervisor Support

Individual innovative behavior is a rich and complicated construct that has been defined and operationalized by different researchers. According to the early theorists, individual innovative behavior is the intentional initiation and implementation of new ideas, processes, and practices (Janssen, 2000). More recently, it has been defined as the behavior of generating and adopting new ideas, and making further efforts to realize the ideas (Lukes and Stephan, 2017). Innovative behavior was initially measured as a unidimensional construct (Scott and Bruce, 1994) before various stages of the innovation process were identified (De Jong and Den Hartog, 2010). For example, Janssen (2000) put forward a three-dimension model including idea generation, idea promotion, and idea realization, while De Jong and Den Hartog (2010) proposed a five-dimension model consisting of idea exploration, idea generation, idea championing, idea implementation, and innovative output. To capture the richness and multidimensionality of the construct, Lukes and Stephan (2017) conceptualized a seven-dimension model, including idea generation, idea search, idea communication, implementation starting activities, involving others, overcoming obstacles, and innovation outputs.

Individual innovative behavior has been thoroughly examined in the workplace (Kwon and Kim, 2020; Li et al., 2020). Empirical evidence has suggested that individual innovative behavior can be influenced by various external and internal factors (Hammond et al., 2011), of which supervisor or managerial support exerts the most proximal influence (Lukes and Stephan, 2017). In a business setting, supervisors or managers contribute to the overall innovation process of employees by providing sufficient and appropriate support, such as giving guidance, championing an idea, or increasing employees’ interests (Shalley et al., 2016). The support from supervisors or managers in the workplace could not only promote individuals’ self-confidence and engagement in the innovative work process, but also help foster an organizational culture that encourages individuals to generate and implement new ideas (Lukes and Stephan, 2017).

Due to the need to cultivate more highly qualified and innovative talents, individual innovative behavior has received much attention in higher education, as to how it is affected by other factors (Hammond, 2016). Recently, some researchers have applied the construct of individual innovative behavior to studies of graduate students and shown it to be effective in understanding innovation by those students (Su and Zhang, 2020). Similar to the findings of early workplace studies, supervisors play a pivotal role in enhancing graduate students’ innovative behavior in a research context (Fan et al., 2018). In addition to creating an open and supportive research environment, supervisors could also be supportive in developing and stimulating graduate students’ innovative behavior, such as honing students’ research skills, increasing their pursuit of innovation, and giving encouragement (Gu et al., 2015). Specifically, supervisor support could catalyze graduate students’ learning process and enhance their research and innovation ability, accelerating the process of generating innovative ideas and transforming the ideas into successful research innovation (Fan et al., 2018). The encouragement and reassurance from supervisors could also motivate graduate students to engage in risky and unconventional activities, such as trying new research methods and communicating creative ideas with others (Shouse and Ma, 2015).

Yet there has been little research examining the role of different types of supervisor support in stimulating graduate students’ innovative behavior, especially from a multidimensional perspective of innovative behavior in a research context. Therefore, a primary purpose of this study is to investigate how the various stages of graduate students’ innovative behavior in research are related to their perceptions of different types of supervisor support.

### Creative Self-Efficacy as a Mediator

Creative self-efficacy refers to an individual’s confidence in his or her ability to generate creative ideas or produce creative outcomes (Tierney and Farmer, 2002). Social cognitive theory suggests that an individual’s self-efficacy is derived from four main sources: mastery experience, vicarious experience, social persuasion, and psychological state (Bandura, 2006). It also suggests that individuals are likely to react to environmental factors by adjusting their psychological cognitive factors (e.g., self-efficacy), which subsequently influences their behavior (Bandura, 1997).

Research has indicated that supervisor support is one of the most influential contextual factors in shaping individuals’ creative self-efficacy across different settings (Tierney and Farmer, 2002). In the context of higher education, supervisors help to enhance graduate students’ creative self-efficacy through assistance and support related to the four abovementioned sources of information. For example, graduate students have more access to successful mastery and vicarious experiences when they receive sufficient help from supervisors (Van Dinther et al., 2011). Their perceptions of creative ability and psychological status also tend to be improved by verbal expressions of encouragement, trust, and praise from supervisors (Gu et al., 2015).

Meanwhile, research has consistently identified creative self-efficacy as a key contributor to individual innovative behavior (Tierney and Farmer, 2011; Puente-Diaz, 2016). Recently, the positive influence of creative self-efficacy on graduate students’ creativity and innovation has been investigated, and the results suggest that creative self-efficacy could affect innovative behavior directly as well as mediate the effect of contextual factors on innovative behavior (Gu et al., 2015; Fan et al., 2018).

In summary, supervisor support is conducive to the development of graduate students’ creative self-efficacy. With greater creative self-efficacy, graduate students have more confidence in engaging in adventurous and risky behavior and are more persistent when facing difficulties in the innovation process. Consequently, their innovative behavior is enhanced. However, the relevant research has mainly viewed innovative behavior as a unidimensional construct, and very little research has focused on the multiple stages of graduate students’ innovative behavior. Therefore, based on the literature reviewed, this study aims to explore the relationships between graduate students’ perceived supervisor support and innovative behavior in research, and the mediating role of creative self-efficacy in the research context. It attempts to answer two questions. First, what are the relationships between graduate students’ perceived supervisor support and their innovative behavior in research? Second, does creative self-efficacy exert a mediation effect on the relationships between supervisor support and the different stages of innovative behavior?

## Methodology

### Participants

An anonymous online questionnaire was administered to graduate students from 52 universities on the Chinese mainland in April 2021. The data analysis was based on 996 (79.5%) valid responses (638 females and 358 males) out of 1253 after a careful data screening. Of the sample, 803 (80.6%) were Master’s students and 193 (19.4%) were Doctoral students. The average age was 25.7 years (*SD* = 4.1), and the students ranged from 21 to 48 years old. In terms of disciplinary background, 34.2% of the students were majoring in social sciences and humanities, 36.3% in technology, 13.6% in science, and 15.9% in medicine.

### Instruments

The online questionnaire used in this study had two parts. The first part was designed to collect demographic information about the participants, including their gender, age, and major. The second part was comprised of three scales, namely the Supervisor Support Scale, Innovative Behavior Inventory, and Creative Self-Efficacy Scale. Considering that all of the participants were native Chinese, a standardized back-translation procedure was carried out in the process of scale translation. All scales began with the heading, “In my current research activities, …”

#### Supervisor Support Scale

The Supervisor Support Scale was developed by Overall et al. (2011) to measure Ph.D. students’ perceived supervisor support. The original scales had 31 items in 3 dimensions. Given that a lengthy questionnaire might lead to increased response burden, low response rate, and decreased data integrity (Lavrakas, 2008), in the present study, five items were selected from each dimension based on their adaptability to the Chinese research context. The three dimensions were academic support (e.g., “My supervisor gives me guidance to find relevant literature and research materials”), personal support (e.g., “My supervisor expresses understanding and empathy when I experience difficulties”), and autonomy support (e.g., “My supervisor provides me with choices and options”). The 15 items were scored on a 7-point Likert scale (1 = strongly disagree, 7 = strongly agree).

#### Innovative Behavior Inventory

The Innovative Behavior Inventory, developed by Lukes and Stephan (2017) to assess employees’ innovative behavior in the workplace, was adapted to measure graduate students’ innovative behavior in research. Slight changes were made to take account of the context by replacing “colleagues and business partners” with “others” (e.g., item 4). The adapted scale has 23 items in 7 dimensions: idea generation (3 items, e.g., “I try new ways of doing things at work”), idea search (3 items, e.g., “I try to get new ideas from others”), idea communication (4 items, e.g., “When I have a new idea, I try to get support for it from supervisor”), implementation starting activities (3 items, e.g., “I develop suitable plans and schedules for the implementation of new ideas”), involving others (3 items, e.g., “When I have a new idea, I look for people who are able to push it through”), overcoming obstacles (4 items, e.g., “I do not give up even when others say it cannot be done”), and innovation outputs (3 items, e.g., “Whenever I worked somewhere, I improved something there”). Each item was scored on a 5-point Likert scale (1 = fully disagree, 5 = fully agree).

#### Creative Self-Efficacy Scale

The eight-item Creative Self-Efficacy Scale (Carmeli and Schaubroeck, 2007) was used to assess graduate students’ creative self-efficacy. Sample items were, “I will be able to achieve most of the goals that I have set for myself in a creative way” and “I am confident that I can perform creatively on many different tasks.” All of the items were scored on a 6-point Likert scale (1 = strongly disagree, 6 = strongly agree).

### Data Analysis

SPSS 25.0 and AMOS 24.0 were used for data analysis. First, the construct validity of each scale was tested by conducting confirmatory factor analysis (CFA) using AMOS 24.0, and the descriptive statistics (mean and standard deviation), correlations, and internal reliability of each subscale were calculated using SPSS 25.0. Second, a structural equation model (SEM) with a mediation analysis was constructed to explore the relationships between graduate students’ perceived supervisor support and innovative behavior in research, and the mediation effect of creative self-efficacy. As the literature (Schreiber et al., 2006) suggests, a model is regarded as acceptable when the Comparative Fit Index (CFI) and Tucker-Lewis Index (TLI) are higher than 0.90 and when the root mean square error of approximation (RMSEA) is less than 0.08. The effect sizes were interpreted based on the guidelines (small = 0.10 to <0.20, medium = 0.20 to <0.30, large = ≥0.30) suggested by Gignac and Szodorai (2016). Furthermore, the multi-group analysis was conducted to examine whether the results were consistent across Master’s and Doctoral students.

## Results

### Construct Validity and Reliability

The construct validity of the three scales is presented in Table 1. The CFA results of the Supervisor Support Scale revealed good model fit (χ^2^ = 530.40, *df* = 83, *p* < 0.001, CFI = 0.98, TLI = 0.97, RMSEA = 0.074), with factor loadings ranging from 0.83 to 0.96. The Cronbach’s α coefficients for the three factors (see Table 2) were 0.94 (academic support), 0.95 (personal support), and 0.96 (autonomy support).

**TABLE 1:** Confirmatory factor analysis results for the scales (*N* = 996).

	χ ^2^	χ ^2^/*df*	*p*	*df*	CFI	TLI	RMSEA
Supervisor Support Scale	530.40	6.39	0.000	83	0.98	0.97	0.074
Innovative Behavior Inventory	1,165.80	5.66	0.000	206	0.96	0.94	0.068
Creative Self-Efficacy Scale	39.65	2.83	0.000	14	0.99	0.99	0.043

**TABLE 2:** Reliabilities, correlations, and descriptive statistics (*N* = 996).

	ACS	PES	AUS	IG	IS	IC	ISA	IVO	OO	IO	CSE
Academic support (ACS)	(0.94)										
Personal support (PES)	0.73**	(0.95)									
Autonomy support (AUS)	0.69**	0.88**	(0.96)								
Idea generation (IG)	0.37**	0.44**	0.45**	(0.86)							
Idea search (IS)	0.35**	0.42**	0.45**	0.72**	(0.86)						
Idea communication (IC)	0.43**	0.49**	0.53**	0.71**	0.72**	(0.87)					
Implementation starting activities (ISA)	0.42**	0.48**	0.51**	0.72**	0.74**	0.83**	(0.93)				
Involving others (IVO)	0.45**	0.48**	0.51**	0.64**	0.71**	0.81**	0.81**	(0.90)			
Overcoming obstacles (OO)	0.36**	0.43**	0.42**	0.70**	0.60**	0.70**	0.73**	0.70**	(0.91)		
Innovation outputs (IO)	0.36**	0.39**	0.40**	0.67**	0.55**	0.67**	0.65**	0.62**	0.81**	(0.89)	
Creative self-efficacy (CSE)	0.45**	0.46**	0.46**	0.71**	0.55**	0.61**	0.62**	0.57**	0.68**	0.68**	(0.98)
Mean	5.49	5.93	5.99	4.06	4.19	4.05	4.16	4.14	3.99	3.87	4.45
Standard deviation	1.27	1.08	1.07	0.62	0.56	0.60	0.59	0.60	0.66	0.71	0.95

**p < 0.01 (two-tailed). The Cronbach’s α coefficients were in the parentheses along the diagonal.

The CFA results also suggested that the Innovative Behavior Inventory fit the data well (χ^2^ = 1,165.80, *df* = 206, *p* < 0.001, CFI = 0.96, TLI = 0.94, RMSEA = 0.068), with factor loadings ranging from 0.66 to 0.92. The Cronbach’s α coefficients of the seven factors ranged from 0.86 (idea generation and idea search) to 0.93 (implementation starting activities).

The CFA results for the Creative Self-Efficacy Scale (χ^2^ = 39.65, *df* = 14, *p* < 0.001, CFI = 0.99, TLI = 0.99, RMSEA = 0.043) showed that the data fit of the model was acceptable. The factor loadings ranged from 0.85 to 0.95 and the Cronbach’s α coefficient (see Table 2) was 0.98, indicating good internal consistency.

### Descriptive Statistics and Correlations

The descriptive statistics and correlation matrix are also summarized in Table 2. Autonomy support (*M* = 5.99, *SD* = 1.07) had the highest mean score out of the three factors of supervisor support, whereas academic support (*M* = 5.49, *SD* = 1.27) had the lowest. As for the Innovative Behavior Inventory, idea search (*M* = 4.19, *SD* = 0.56) had the most positive score and innovation outputs (*M* = 3.87, *SD* = 0.71) had the lowest. The mean score and standard deviation of creative self-efficacy were 4.45 and 0.95, respectively, while the correlation matrix indicated that all of the factors of the three scales were significantly and positively correlated with large effect sizes.

### Structural Equation Model Analysis

**FIGURE 1:**
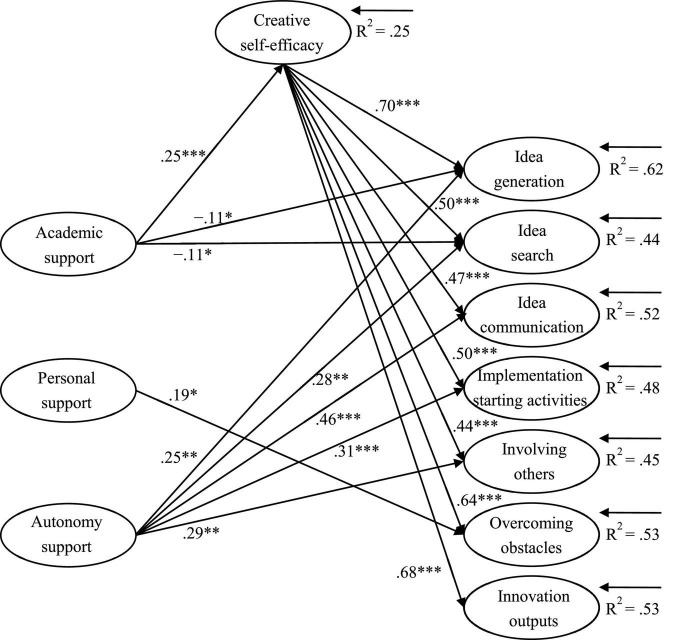
Structural equation model results showing significant path coefficients (*N* = 996). *p < 0.05; ^**^p < 0.01; ^***^p < 0.001; goodness-of-fit indices: χ^2^ = 3,123.00, *df* = 921, *p* < 0.001, CFI = 0.96, TLI = 0.95, RMSEA = 0.049.

Structural equation model was performed to examine the relationships between graduate students’ perceived supervisor support and their innovative behavior in research. The model was based on the assumption that correlations were allowed between the independent variable (supervisor support), the dependent variable (innovative behavior), and the mediator (creative self-efficacy). As shown in Figure 1, the data fit was acceptable (χ^2^ = 3,123.00, *df* = 921, *p* < 0.001, CFI = 0.96, TLI = 0.95, RMSEA = 0.049). The explained variance of the seven factors of innovative behavior ranged from 0.44 (idea search) to 0.62 (idea generation).

The SEM results showed that academic support had a weakly negative association with idea generation (β = −0.11, *p* < 0.05) and idea search (β = −0.11, *p* < 0.05), with small effect sizes. Personal support was positively associated with overcoming obstacles (β = 0.19, *p* < 0.05), with a small effect size. Autonomy support had a significantly positive association with idea generation (β = 0.25, *p* < 0.01), idea search (β = 0.28, *p* < 0.01), idea communication (β = 0.46, *p* < 0.001), implementation starting activities (β = 0.31, *p* < 0.001), and involving others (β = 0.29, *p* < 0.01), with moderate to large effect sizes.

The results of the mediation analysis (Figure 1) suggested that academic support from the supervisor (β = 0.25, *p* < 0.001) had a positive association with creative self-efficacy, with a moderate effect size. The graduate students’ creative self-efficacy was positively associated with all of the factors of innovative behavior, with large effect sizes.

To further test whether the results were consistent across Master’s and Doctoral students, a multi-group analysis was conducted following Byrne’s (2010) guidelines. First, the configural invariance was tested and the results indicated that the configural model had an acceptable model fit (χ^2^ = 4,784.66, *df* = 1,842, *p* < 0.001, CFI = 0.95, TLI = 0.94, RMSEA = 0.040), suggesting that the baseline model has an acceptable fit across Master’s and Doctoral students. Second, the measurement and structural invariance were examined by comparing the differences in CFI values (ΔCFI). According to Cheung and Rensvold (2002), there was a significant difference between the models if the ΔCFI values were greater than 0.01. The results in Table 3 showed that ΔCFI values didn’t reach the criteria, indicating that the measurement and structural models were operating equivalently across Master’s and Doctoral students.

**TABLE 3:** Results of multi-group analysis across Master’s and Doctoral students.

Model	Model fitness								Model comparison		
	χ ^2^	*df*	*p*	TLI	CFI	RMSEA	AIC	ECVI	Δχ ^2^	Δ *df*	Δ CFI
Configural model	4,784.66	1,842	0.000	0.94	0.95	0.040	5,424.66	5.46	–	–	–
Measurement weights	4,835.92	1,877	0.000	0.94	0.95	0.040	5,405.92	5.44	51.26	35	−0.001
Structural weights	4,882.56	1,908	0.000	0.94	0.95	0.040	5,390.56	5.42	46.64	31	0.000
Structural covariances	4,902.79	1,914	0.000	0.94	0.95	0.040	5,398.79	5.43	20.23	6	0.000
Structural residuals	5,036.86	1,943	0.000	0.94	0.94	0.040	5,474.86	5.51	134.07	29	−0.002
Measurement residuals	5,488.01	2,002	0.000	0.93	0.94	0.040	5,808.01	5.84	451.15	59	−0.008

*Master’s students n = 803, Doctoral students n = 193; Δχ^2^, Δdf, and ΔCFI represent the differences in χ^2^ values, degrees of freedom and CFI values between each hierarchical model.*

### Mediation Analysis

To test if creative self-efficacy exerts a mediation effect on the relationships between supervisor support and the different stages of innovative behavior, a mediation analysis based on 2,000 bootstrap samples was conducted. According to Hayes (2009), the indirect effect is significant when zero does not lie between the lower and upper bounds of the 95% confidence interval. As summarized in Table 4, the graduate students’ creative self-efficacy mediated the relationships between academic support and innovative behavior in research, with small effect sizes. However, the mediation effect of creative self-efficacy was not significant between the other two factors of supervisor support and innovative behavior in research.

**TABLE 4:** The estimates of direct effects and indirect effects of the 95% confidence intervals.

				95% CIs		
Dependent variable	Independent variable	Direct effect	Indirect effect	Lower 2.5%	Upper 2.5%	*R* ^2^
Idea generation	Academic support	–0.11	0.18**	0.09	0.27	0.62
	Personal support	0.01	0.12	–0.08	0.31	
	Autonomy support	0.25	0.08	–0.09	0.26	
Idea search	Academic support	–0.11	0.13**	0.07	0.20	0.44
	Personal support	0.07	0.09	–0.05	0.22	
	Autonomy support	0.28	0.06	–0.07	0.18	
Idea communication	Academic support	–0.03	0.12**	0.06	0.18	0.52
	Personal support	–0.07	0.08	–0.05	0.22	
	Autonomy support	0.46	0.06	–0.06	0.18	
Implementation starting activities	Academic support	–0.03	0.13**	0.07	0.19	0.48
	Personal support	0.01	0.09	–0.05	0.22	
	Autonomy support	0.31	0.06	–0.06	0.19	
Involving others	Academic support	0.07	0.11**	0.06	0.17	0.45
	Personal support	–0.02	0.08	–0.04	0.21	
	Autonomy support	0.29	0.05	–0.05	0.17	
Overcoming obstacles	Academic support	–0.07	0.16**	0.09	0.24	0.53
	Personal support	0.19	0.11	–0.07	0.28	
	Autonomy support	0.02	0.08	–0.08	0.24	
Innovation outputs	Academic support	–0.03	0.17**	0.09	0.26	0.53
	Personal support	0.03	0.12	–0.07	0.30	
	Autonomy support	0.10	0.08	–0.08	0.25	

**p < 0.01. Bold items showing significant mediation effect.

## Discussion

The present study fills the gap in the knowledge of how different stages of graduate students’ innovative behavior in research are related to their perceptions of various types of supervisor support, especially in the Chinese context. It contributes to the literature by offering empirical evidence on the meditation of creative self-efficacy in the relationships between supervisor support and innovative behavior in research, particularly the role of creative self-efficacy in offsetting the negative effect of academic support. The study also tested and strengthened the multidimensional Innovative Behavior Inventory in the research context by using a sample of Chinese graduate students, which extended the applicability of the scale in new contexts.

### Relationships Between Graduate Students’ Perceived Supervisor Support and Innovative Behavior in Research

The SEM results showed that personal support was positively associated with overcoming obstacles, indicating that graduate students are more likely to overcome difficulties and challenges in research innovation when their supervisors are emotionally supportive and friendly. This finding is in line with studies suggesting that personal support is conducive to individuals’ creativity and innovation (Tierney and Farmer, 2002; Fan et al., 2018). Encouragement and reassurance from supervisors can not only increase graduate students’ research motivation and engagement (Ruzek et al., 2016), but it can also help foster a free and open research environment where expanded sources tend to be available for graduate students to identify and cope with the challenges and difficulties in research (Overall et al., 2011). Empirical research has further suggested graduate students whose supervisors show the appropriate interest and respect are more confident in their knowledge and abilities (Hughes et al., 2008), which usually leads to the application of more adaptive coping strategies when facing obstacles in the innovation process (Overall et al., 2011). Researchers have also argued that graduate students and supervisors exchange information more efficiently when the former are emotionally well supported by the latter (Tschannen-Moran, 2001). The exchange of information and knowledge may help graduate students overcome certain obstacles in research innovation (Gu et al., 2014).

The results also showed that autonomy support was positively associated with idea generation, idea search, idea communication, implementation starting activities, and involving others. This is in line with the findings of research on employees, which indicate that individuals are more likely to engage in innovative behavior when they are encouraged by supervisors to make decisions on their own (Lu et al., 2012; Al-Husseini and Elbeltagi, 2014). The positive associations may be related to autonomy support, which aims to provide graduate students with the freedom to try new things and take their own initiatives (Overall et al., 2011), thus spontaneously facilitating the innovation process (Parker et al., 2001). However, the non-significant relationship between autonomy support and the other innovative behavior was somewhat inconsistent with the findings of prior research. Coelho and Augusto (2010) found that a positive relationship between perceived autonomy and innovation existed only when individuals had high task identity and received rich feedback. Therefore, low task identity and a lack of feedback could severely limit individuals’ knowledge and skill development and undermine their intrinsic motivation. Accordingly, the effect of autonomy support on innovation might be eroded.

Previous studies of both employees and graduate students have suggested that intellectual guidance and academic support could enhance individuals’ creativity and innovation, including helping to generate novel ideas and think in an exploratory fashion (Bass and Riggio, 2006; Fan et al., 2018). However, in this study, academic support was negatively associated with idea generation and idea search, indicating that the more academic help received from supervisors, the less likely graduate students are to generate and search for novel ideas. Delamont et al. (1998) suggested that graduate students may feel that they lack independence and are overcontrolled when they perceive the degree of intervention in their academic or research activities to be interference. The direct and excessive support from supervisors may also weaken graduate students’ autonomous beliefs by shaping a research environment where students may experience decreased motivation, low level of self-confidence, and a feeling of being overcontrolled (Overall et al., 2011; Fan et al., 2018). Tight control of research-related matters, such as setting out a clear-cut routine and deciding on research topics for graduate students, could limit their initiative and originality, inhibiting the generation of and search for novel ideas (Lessing and Schulze, 2002).

It is also worth noting that, of the three factors of graduate students’ perceived supervisor support, the mean score of academic support was the lowest and that of autonomy support was the highest. In China, the major responsibility of supervisors in the new era is to develop graduate students’ research and innovative ability and prepare them for being independent and innovative researchers and practitioners (Peng, 2015). As a result, compared with the direct help from supervisors to complete research activities, graduate students might perceive more encouragement and opportunities in terms of conducting research independently and acting on their own decisions.

### Creative Self-Efficacy as a Mediator of the Relationships Between Graduate Students’ Perceived Supervisor Support and Innovative Behavior in Research

This study expands the knowledge of the relationships between graduate students’ perceived supervisor support and their innovative behavior in research. Compared with the negative and non-significant direct relationships between academic support and innovative behavior found in this study, the mediation analysis revealed that creative self-efficacy mediated the influence of academic support on all stages of graduate students’ innovative behavior, with small effect sizes. The findings indicate that the effects of supervisor support on graduate students’ innovative behavior are significantly actualized through their increased creative self-efficacy. This is consistent with the findings of previous research that graduate students with higher creative self-efficacy tend to be more dedicated to creativity and innovation when they receive sufficient academic guidance and help from supervisors (Gu et al., 2015; Tierney and Lanford, 2016). When graduate students feel more confident in their creative abilities, supervisors’ research knowledge and expertise play a bigger role in enhancing their research abilities, persistence, and willingness to pursue novel efforts (Choi, 2004), facilitating the development of innovative behavior (Puente-Diaz, 2016).

Although personal support and autonomy support had direct positive associations with certain aspects of innovative behavior in research, the results of the mediation analysis showed that creative self-efficacy had a non-significant mediation effect on the relationships. Correspondingly, personal and autonomy support had null associations with the graduate students’ creative self-efficacy, highlighting the significant direct effect of these two types of support on innovative behavior in research. Nevertheless, as limited research has been conducted on supervisor support in increasing graduate students’ creative self-efficacy, future studies should reveal the potential relationships between the variables, especially in the research context.

### Limitations

This study was designed to offer insights into the relationships between supervisor support and graduate students’ innovative behavior in research. Several limitations should be pointed out to set the direction for future studies. First, the design of the current study was cross-sectional, which is insufficient to explore the consistent causal relationships between supervisor support and graduate students’ innovative behavior. Therefore, a longitudinal study is expected to examine the causal relationships between the variables. Second, response bias may exist as the research data were based on graduate students’ self-report measures. To collect more objective and accurate data, future studies are encouraged to use more carefully designed measurements with objective questions (Cheung and Chan, 2002). Third, the SEM mediation analysis in the present study was insufficient to determine the threshold at which supervisor support changes from being productive regarding creative self-efficacy to being detrimental, and this could be further explored in the near future. Last but not least, this study did not further explore the potential differences in the measurement and structural models across graduate students of different disciplines, and future study may consider addressing the issue by multi-group analysis.

## Implications

In this study, the relationships between graduate students’ perceived supervisor support and innovative behavior in research were explored, as well as the mediating role of creative self-efficacy in those relationships. The results indicate that supervisor support and creative self-efficacy are both important factors in developing graduate students’ innovative behavior in research, offering some important implications for enhancing graduate students’ research innovation.

The positive relationship between personal support and overcoming obstacles indicates that graduate students’ perceptions of the emotional support, respect, and concern from supervisors could help them overcome difficulties and challenges during research innovation and facilitate the development of their innovative behavior in research. These results highlight the importance and need for supervisors to care more about graduate students’ emotional well-being. A warm attitude and friendly behavior, such as being supportive of students struggling with setbacks and offering compliments when they perform well, are expected from supervisors. In addition, HEIs could help construct a collaborative research environment and provide opportunities, such as interactive seminars, to secure an emotional connection between supervisors and graduate students.

The positive relationships between autonomy support and multiple stages of innovative behavior indicate that graduate students’ research innovation is closely related to the freedom and control they perceive in their own research. Apart from supervisors being fully aware of the importance of encouraging graduate students to express their ideas and act on their own decisions, they could provide the practical assistance and guidance essential for graduate students to become autonomous and independent researchers during supervision. Meanwhile, HEIs could try to build a free and open research environment to encourage graduate students to take initiative in their own research. The results further suggest that appropriate feedback and high task identity are significant in developing independent and innovative researchers. Therefore, graduate students could be offered more opportunities and multiple channels through which to receive feedback and bolster their confidence in finishing research tasks, such as regular meetings with supervisors, research projects, academic lectures, and innovative practices.

Given that academic support had negative and non-significant relationships with innovative behavior, the detrimental effect, particularly of excessive academic support on innovative behavior, should be noted by HEIs and supervisors. It is important for supervisors to rein in their power over students’ research and strike a balance between directly helping students in research-related activities and offering them the chance to conduct research independently. More importantly, the mediating effect of creative self-efficacy suggests that improving graduate students’ confidence in their creative abilities may considerably enhance the beneficial effect of academic support on innovative behavior. Therefore, HEIs, supervisors, and researchers should attach great importance to improving graduate students’ belief in their own creative potential. This could be achieved by facilitating the successful completion of students’ research tasks, convincing them to think and act creatively during instruction and supervision, and establishing supervisors or renowned researchers as role models for them to emulate.

## Data Availability Statement

The raw data supporting the conclusions of this article will be made available by the authors, without undue reservation.

## Ethics Statement

The studies involving human participants were reviewed and approved by the Shandong University. The patients/participants provided their written informed consent to participate in this study.

## Author Contributions

JH and NL drafted the manuscript. FW revised and finalized the manuscript. All authors contributed to the article and approved the submitted version.

## Conflict of Interest

The authors declare that the research was conducted in the absence of any commercial or financial relationships that could be construed as a potential conflict of interest.

## Publisher’s Note

All claims expressed in this article are solely those of the authors and do not necessarily represent those of their affiliated organizations, or those of the publisher, the editors and the reviewers. Any product that may be evaluated in this article, or claim that may be made by its manufacturer, is not guaranteed or endorsed by the publisher.
